# Crystal structure of *N*,*N*′-bis­(2,4-di­fluoro­benzo­yloxy)benzene-1,2:4,5-tetra­carboximide

**DOI:** 10.1107/S2056989018001226

**Published:** 2018-01-26

**Authors:** Sandra Fusco, Angela Tuzi, Roberto Centore, Antonio Carella

**Affiliations:** a Dipartimento di Scienze Chimiche, Università degli Studi di Napoli ’Federico II’, Complesso di Monte S. Angelo, Via Cinthia, 80126 Napoli, Italy

**Keywords:** crystal structure, imide, weak hydrogen bond, fluorine

## Abstract

Mol­ecules of the title compound, C_24_H_8_F_4_N_2_O_8_, have *C*_i_ point-group symmetry in the crystal, as they lie on crystallographic inversion centres (*Z*′ = 1/2). The di­fluoro­phenyl ring is disordered over two orientations; the final refined occupancy factors of the two components of disorder are 0.947 (4) and 0.053 (4). In the crystal, some C_ar_—H⋯F inter­actions are present, which involve the most acidic H atom of the mol­ecule.

## Abstract 2

The title compound crystallizes with half a mol­ecule in the asymmetric unit and shows C_ar_—H⋯F inter­actions in the crystal packing.

## Chemical context   

Heterocycles are key compounds in synthetic chemistry. In addition to their applications in drugs, bioactive and tautomeric compounds (D’Errico *et al.*, 2012 ▸; Piccialli *et al.*, 2007 ▸; Centore *et al.*, 2013 ▸), aromatic heterocycles play an important role in modern materials chemistry, because they are used as building blocks of active mol­ecules in many emerging fields of advanced materials, for example, conducting polymers (Heeger, 2010 ▸), organic field-effect transistors (Miao, 2014 ▸), organic solar cells (Nielsen *et al.*, 2015 ▸), liquid crystals (Centore *et al.*, 1996 ▸) and nonlinear optically active compounds (Carella *et al.*, 2007 ▸; Centore *et al.*, 1999 ▸).

Aromatic di­imides, in particular, are a class of heterocyclic compounds well known for their outstanding properties as *n*-type organic semiconductors. Very high electron mobilities have been measured for perylenedi­imides (Schmidt *et al.*, 2007 ▸) and naphthalenedi­imides (Yan *et al.*, 2009 ▸). The research on *n*-type organic semiconductors has also shown that electron mobilities and device performances can be improved by extensive replacement of H atoms by fluorine (Facchetti *et al.*, 2003 ▸).

Following these issues, we report here the structural investigation of the title compound, *N*,*N*′-bis­(2,4-di­fluoro­benzo­yloxy)benzene-1,2:4,5-tetra­carboximide, which is a fluorinated derivative of the simplest aromatic bis­(imide).
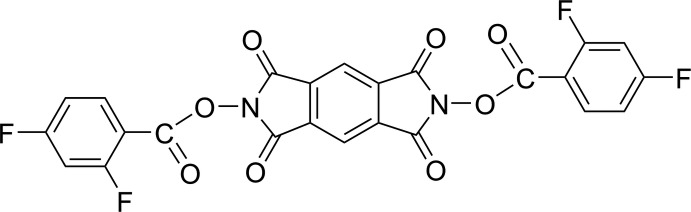


## Structural commentary   

**Figure 1:**
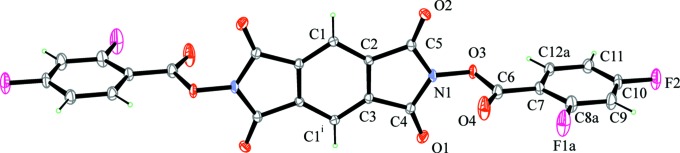
The mol­ecular structure of the title compound, with displacement ellipsoids drawn at the 30% probability level. Only the most populated orientation of the disordered di­fluoro­phenyl ring is shown. [Symmetry code: (i) −*x* + 1, −*y*, −*z* + 1.]

Mol­ecules of the title compound in the crystal lie on crystallographic inversion centres and have *C*_i_ point-group symmetry. Thus, the asymmetric unit is formed by half a mol­ecule, as shown in Fig. 1 ▸. The di­fluoro­phenyl ring is disordered over two orientations that differ by a rotation of 180° around the C6—C7 bond. The atomic positions for the two orientations of the di­fluoro­phenyl ring are completely superimposed for all atoms, except for the *ortho*-F atom, for which split positions were observed. The final refined occupancy factors of the two components of disorder are 0.947 (4) and 0.053 (4). Mol­ecules adopt a nonplanar conformation, mainly because of a torsion around the O3—N1 bond. In particular, the pentaa­tomic ring (atoms C2/C3/C4/N1/C5) is planar within 0.0164 (14) Å, while the phenyl ring (C7/C8a/C9/C10/C11/C12a) is planar within 0.002 (2) Å. The dihedral angle between the mean planes of the two rings is 86.14 (8)°.

The C6—O3 bond length [1.402 (3) Å] is significantly longer than the mean value for esters of aromatic acids (1.337 Å; Allen *et al.*, 1987 ▸). This suggests a reduced contribution of the minor resonance form of the ester group, in which one of the lone pairs of the alk­oxy oxygen forms a double bond with the carbonyl C atom that breaks its double bond with the other O atom, thereby giving it a negative charge. This, in turn, can be due to the presence of the electronegative N atom bonded to O3.

## Supra­molecular features   

There are weak hydrogen-bond donors (C_ar_—H) and strong hydrogen-bond acceptors (carbonyl O atoms) in the title compound. Moreover, F atoms are present as well, whose low hydrogen-bonding-acceptor capability, if any, has been the subject of a long debate in the literature (Dunitz & Taylor, 1997 ▸; Dunitz, 2004 ▸). Actually, it is now established that the C—H⋯F inter­action is generally weak and does not play a significant structural role in crystal packing when stronger and more polarizable acceptors than the C—F group are present. On the other hand, when the carbon acidity is suitably enhanced, and in the absence of competing acceptors, the (weak) hydrogen-bonding nature of the C—H⋯F inter­action is revealed (Thalladi *et al.*, 1998 ▸).

**Figure 2:**
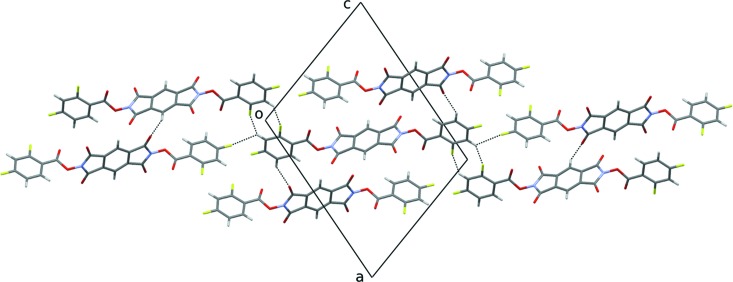
Partial crystal packing of the title compound, showing the C—H⋯F and C—H⋯O inter­actions as dashed lines. Only the most populated orientation of the disordered di­fluoro­phenyl ring is shown.

The most acidic C_ar_—H group in the title compound is C9—H, because it has two *ortho* C—F-group neighbours. It is involved in weak hydrogen-bonding inter­actions with fluorine, as shown in Fig. 2 ▸ and Table 1 ▸. In particular, C9—H acts as bifurcated donor to the F1*A* and F2 atoms. In the first case, 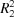
(8) ring motifs are formed across inversion centres. In the second case, chain patterns running parallel to *a* − *b* + *c*/2 are formed. These patterns are quite similar to the supra­molecular synthons **II** and **IV** reported in the Scheme 2 of Thalladi *et al.* (1998 ▸). It is quite remarkable that significant C—H⋯F inter­actions are only given by C9—H, which is the most acidic H atom of the mol­ecule.

Other acidic H atoms are C11—H, because it has one *ortho* C—F group, and C1—H. They are involved in weak hydrogen-bonding inter­actions with the O1 and O2 carbonyl acceptors, respectively, see Table 1 ▸ and Fig. 2 ▸.

## Database survey   

A search of the Cambridge Structural Database (CSD, Version 5.38, last update May 2017; Groom *et al.*, 2016 ▸) gave no match for the title compound. We have searched for *N*-oxycarbonyl­imides using two filters (three-dimensional coordinates determined and not disordered). 47 hits were found. Within this set, 40 hits are *N*-oxycarbonyl derivatives of succinimide and 6 hits are derivatives of phthalimide. Here follows the full list of refcodes for the CSD search: ADEFUL, AFUXEE, ALOPAU, AWUXOF, BANTOA, CILBUV, COZFOM, DOFTEZ, EABXIO, EABXIO01, FUPPEM, GULRAI, GUVCAB, ICEWIY, LOZFAH, MAMDOU, MILFOE, MIPHUP, MIZJEM, MOZYOQ, NANWUU, OQUPOG, PIGKIZ, SEZWIE, SOSDEK, SUDJIM, SUDWUL, SUDXAS, SUDXAS01, TEQDEY, TUJRIB, UJAFER, UJUBOS, UJUBUY, VALSUZ, WALPEH, WIDKEB, YAFMEY, YAFPOL, YAGBEP, YUJZIN, YUJZOT, ZEPSES, ZEPSIW, ZOQQOL, ZOQQUR, ZALKUV.

**Figure 3:**
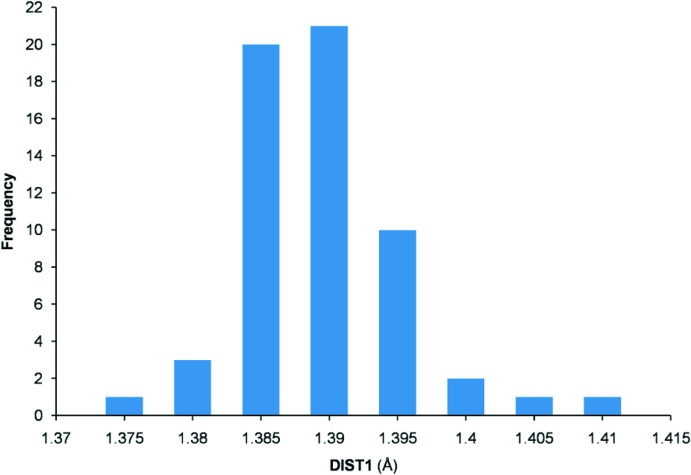
Histogram of the N—O bond lengths (DIST1) in the 47 *N*-oxycarbonyl­imide hits found in the CSD search.

The hits found are crystal structures determined at temperatures in the range 90–298 K. In the 47 hits, the N1—O3 distance (DIST1) ranges between 1.375 and 1.408 Å, with an average value of 1.388 (6) Å. On the other hand, the distance O3—C6 (DIST2) is between 1.350 and 1.423 Å, with an average value of 1.393 (15) Å. The histogram of the distribution of DIST1 over the 47 hits found is shown in Fig. 3 ▸. The values of DIST1 and DIST2 found in the title compound [N1—O3 = 1.381 (2) Å and O3—C6 = 1.402 (3) Å] are fully consistent with the average values determined from the 47 hits.

## Synthesis and crystallization   

*N*,*N*′-Di­hydroxy­benzene-1,2:4,5-tetra­carboximide (Centore & Carella, 2013 ▸) (1.000 g, 4.030 mmol) was suspended in 20 ml of dry pyridine and the system was kept under stirring at room tem­perature. 2,4-Di­fluoro­benzoyl­chloride (1.991 g, 11.28 mmol) was added dropwise and the previous suspension turned into a dark solution. The solution was warmed and boiled gently for 45 min. Absolute ethanol (2 ml) and, after 2 min, distilled water (0.2 ml) were then added and the system cooled slowly to room temperature and filtered. The white crystals were washed on the filter with absolute ethanol. From the recovered material it was possible to isolate several single crystals suitable for X-ray analysis. The yield was 55% (m.p. 604 K).

## Refinement   

Crystal data, data collection and structure refinement details are summarized in Table 2 ▸. The H atoms were generated stereochemically and were refined by the riding model. For all H atoms, *U*_iso_(H) = 1.2*U*_eq_(carrier). The di­fluoro­phenyl ring is disordered over two orientations, which differ by a rotation of 180° around the phenyl to carbonyl bond. Split positions were only observed for the *ortho*-F atom. The two split positions were refined by applying SADI restraints on bond lengths. The final refined occupancy factors of the two components of disorder are 0.947 (4) and 0.053 (4).

## Supplementary Material

Crystal structure: contains datablock(s) global, I. DOI: 10.1107/S2056989018001226/fy2124sup1.cif

Structure factors: contains datablock(s) I. DOI: 10.1107/S2056989018001226/fy2124Isup2.hkl

CCDC reference: 1818213

Additional supporting information:  crystallographic information; 3D view; checkCIF report

## Figures and Tables

**Table 1:** Hydrogen-bond geometry (Å, °)

*D*—H⋯*A*	*D*—H	H⋯*A*	*D*⋯*A*	*D*—H⋯*A*
C1—H1⋯O2^i^	0.95	2.37	3.271 (3)	158
C9—H9⋯F1*A* ^ii^	0.95	2.51	3.287 (3)	139
C9—H9⋯F2^iii^	0.95	2.63	3.307 (3)	129
C11—H11⋯O1^iv^	0.95	2.39	3.200 (3)	143

Symmetry codes: (i) 
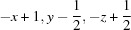
; (ii) 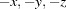; (iii) 
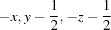
; (iv) 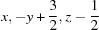.

**Table 2:** Experimental details

Crystal data	
Chemical formula	C_24_H_8_F_4_N_2_O_8_
*M*_r_	528.32
Crystal system, space group	Monoclinic, *P*2_1_/*c*
Temperature (K)	173
*a*, *b*, *c* (Å)	17.100 (6), 4.744 (2), 13.662 (4)
β (°)	106.83 (2)
*V* (Å^3^)	1060.8 (7)
*Z*	2
Radiation type	Mo *K*α
μ (mm^−1^)	0.15
Crystal size (mm)	0.40 × 0.15 × 0.01

Data collection	
Diffractometer	Bruker–Nonius KappaCCD
Absorption correction	Multi-scan (*SADABS*; Bruker, 2001 ▸)
*T*_min_, *T*_max_	0.931, 0.986
No. of measured, independent and observed [*I* > 2σ(*I*)] reflections	7948, 2396, 1440
*R*_int_	0.061
(sin θ/λ)_max_ (Å^−1^)	0.650

Refinement	
*R*[*F* ^2^ > 2σ(*F* ^2^)], *wR*(*F* ^2^), *S*	0.048, 0.120, 1.05
No. of reflections	2396
No. of parameters	176
No. of restraints	1
H-atom treatment	H-atom parameters constrained
Δρ_max_, Δρ_min_ (e Å^−3^)	0.26, −0.32

Computer programs: *COLLECT* (Nonius, 1999 ▸), *DIRAX/LSQ* (Duisenberg *et al.*, 2000 ▸), *EVALCCD* (Duisenberg *et al.*, 2003 ▸), *SIR97* (Altomare *et al.*, 1999 ▸), *SHELXL2016* (Sheldrick, 2015 ▸), *ORTEP-3 for Windows* and *WinGX* (Farrugia, 2012 ▸) and *Mercury* (Macrae *et al.*, 2008 ▸).

## supplementary crystallographic information

### Crystal data

**Table:**

C_24_H_8_F_4_N_2_O_8_	*F*(000) = 532
*M**_r_* = 528.32	*D*_x_ = 1.654 Mg m^−^^3^
Monoclinic, *P*2_1_/*c*	Mo *K*α radiation, λ = 0.71073 Å
*a* = 17.100 (6) Å	Cell parameters from 85 reflections
*b* = 4.744 (2) Å	θ = 5.1–21.4°
*c* = 13.662 (4) Å	µ = 0.15 mm^−^^1^
β = 106.83 (2)°	*T* = 173 K
*V* = 1060.8 (7) Å^3^	Plate, colourless
*Z* = 2	0.40 × 0.15 × 0.01 mm

### Data collection

**Table:**

Bruker–Nonius KappaCCD diffractometer	2396 independent reflections
Radiation source: normal-focus sealed tube	1440 reflections with *I* > 2σ(*I*)
Graphite monochromator	*R*_int_ = 0.061
Detector resolution: 9 pixels mm^-1^	θ_max_ = 27.5°, θ_min_ = 3.4°
CCD rotation images, thick slices scans	*h* = −22→21
Absorption correction: multi-scan (*SADABS*; Bruker, 2001)	*k* = −6→6
*T*_min_ = 0.931, *T*_max_ = 0.986	*l* = −15→17
7948 measured reflections	

### Refinement

**Table:**

Refinement on *F*^2^	Primary atom site location: structure-invariant direct methods
Least-squares matrix: full	Secondary atom site location: difference Fourier map
*R*[*F*^2^ > 2σ(*F*^2^)] = 0.048	Hydrogen site location: inferred from neighbouring sites
*wR*(*F*^2^) = 0.120	H-atom parameters constrained
*S* = 1.05	*w* = 1/[σ^2^(*F*_o_^2^) + (0.045*P*)^2^ + 0.4397*P*] where *P* = (*F*_o_^2^ + 2*F*_c_^2^)/3
2396 reflections	(Δ/σ)_max_ < 0.001
176 parameters	Δρ_max_ = 0.26 e Å^−^^3^
1 restraint	Δρ_min_ = −0.32 e Å^−^^3^

### Special details

**Table:**

Geometry. All esds (except the esd in the dihedral angle between two l.s. planes) are estimated using the full covariance matrix. The cell esds are taken into account individually in the estimation of esds in distances, angles and torsion angles; correlations between esds in cell parameters are only used when they are defined by crystal symmetry. An approximate (isotropic) treatment of cell esds is used for estimating esds involving l.s. planes.
Refinement. The difluorophenyl ring is disordered over two orientations. The two split positions were refined by applying SADI restraints on bond lengths.

### Fractional atomic coordinates and isotropic or equivalent isotropic displacement parameters (Å^2^)

**Table:**

	*x*	*y*	*z*	*U*_iso_*/*U*_eq_	Occ. (<1)
C1	0.53428 (13)	−0.2108 (5)	0.44840 (16)	0.0196 (5)	
H1	0.557147	−0.349497	0.414668	0.024*	
C2	0.47095 (12)	−0.0358 (5)	0.39704 (15)	0.0177 (5)	
C3	0.43844 (12)	0.1672 (5)	0.44705 (16)	0.0191 (5)	
C4	0.37402 (13)	0.3243 (5)	0.36996 (17)	0.0226 (5)	
C5	0.42706 (13)	−0.0236 (5)	0.28530 (16)	0.0208 (5)	
C6	0.24590 (14)	0.1960 (6)	0.15987 (18)	0.0306 (6)	
C7	0.19802 (14)	0.3233 (6)	0.06271 (17)	0.0260 (5)	
C9	0.07036 (16)	0.3516 (7)	−0.0723 (2)	0.0406 (7)	
H9	0.015390	0.292807	−0.101232	0.049*	
C10	0.10527 (16)	0.5453 (7)	−0.11934 (18)	0.0354 (7)	
C11	0.18428 (15)	0.6341 (6)	−0.08085 (19)	0.0333 (6)	
H11	0.206836	0.769181	−0.116313	0.040*	
C8A	0.11781 (16)	0.2431 (6)	0.0194 (2)	0.0369 (7)	0.947 (4)
F1A	0.08458 (11)	0.0511 (5)	0.06620 (15)	0.0714 (8)	0.947 (4)
C12A	0.23029 (14)	0.5223 (6)	0.01072 (18)	0.0289 (6)	0.947 (4)
H12A	0.285205	0.582553	0.038893	0.035*	0.947 (4)
C8B	0.11781 (16)	0.2431 (6)	0.0194 (2)	0.0369 (7)	0.053 (4)
H8B	0.094436	0.108079	0.053933	0.044*	0.053 (4)
C12B	0.23029 (14)	0.5223 (6)	0.01072 (18)	0.0289 (6)	0.053 (4)
F1B	0.3060 (9)	0.615 (6)	0.037 (2)	0.043*	0.053 (4)
F2	0.05967 (10)	0.6562 (4)	−0.20823 (12)	0.0554 (6)	
N1	0.37352 (12)	0.1997 (5)	0.27779 (14)	0.0256 (5)	
O1	0.33327 (10)	0.5199 (4)	0.38057 (13)	0.0315 (4)	
O2	0.43410 (10)	−0.1664 (4)	0.21582 (12)	0.0258 (4)	
O3	0.32607 (9)	0.2995 (4)	0.18452 (11)	0.0268 (4)	
O4	0.22619 (12)	0.0326 (6)	0.21309 (16)	0.0623 (8)	

### Atomic displacement parameters (Å^2^)

**Table:**

	*U*^11^	*U*^22^	*U*^33^	*U*^12^	*U*^13^	*U*^23^
C1	0.0185 (10)	0.0213 (12)	0.0195 (10)	0.0013 (10)	0.0062 (8)	0.0007 (10)
C2	0.0187 (10)	0.0186 (12)	0.0162 (9)	−0.0022 (9)	0.0056 (8)	0.0006 (9)
C3	0.0162 (10)	0.0209 (12)	0.0195 (10)	−0.0013 (9)	0.0040 (8)	0.0037 (10)
C4	0.0197 (10)	0.0247 (13)	0.0220 (11)	0.0006 (10)	0.0038 (8)	0.0030 (11)
C5	0.0214 (10)	0.0206 (12)	0.0189 (10)	−0.0006 (10)	0.0037 (8)	0.0034 (10)
C6	0.0236 (12)	0.0388 (16)	0.0262 (12)	−0.0060 (12)	0.0023 (10)	0.0058 (12)
C7	0.0241 (11)	0.0309 (14)	0.0197 (11)	−0.0020 (11)	0.0012 (9)	0.0036 (11)
C9	0.0254 (12)	0.055 (2)	0.0326 (14)	−0.0102 (13)	−0.0051 (10)	0.0056 (14)
C10	0.0307 (13)	0.0479 (18)	0.0209 (11)	0.0052 (13)	−0.0034 (10)	0.0070 (13)
C11	0.0286 (12)	0.0401 (17)	0.0294 (13)	−0.0004 (12)	0.0055 (10)	0.0120 (12)
C8A	0.0308 (13)	0.0423 (17)	0.0331 (14)	−0.0113 (13)	0.0021 (11)	0.0098 (13)
F1A	0.0388 (10)	0.0959 (19)	0.0636 (13)	−0.0361 (11)	−0.0101 (9)	0.0451 (13)
C12A	0.0209 (11)	0.0368 (16)	0.0262 (11)	−0.0034 (11)	0.0021 (9)	0.0042 (12)
C8B	0.0308 (13)	0.0423 (17)	0.0331 (14)	−0.0113 (13)	0.0021 (11)	0.0098 (13)
C12B	0.0209 (11)	0.0368 (16)	0.0262 (11)	−0.0034 (11)	0.0021 (9)	0.0042 (12)
F2	0.0409 (9)	0.0744 (14)	0.0363 (9)	−0.0026 (9)	−0.0119 (7)	0.0227 (9)
N1	0.0271 (10)	0.0289 (12)	0.0162 (9)	0.0073 (9)	−0.0010 (7)	0.0046 (9)
O1	0.0284 (9)	0.0306 (10)	0.0324 (9)	0.0103 (8)	0.0037 (7)	0.0007 (8)
O2	0.0313 (9)	0.0267 (10)	0.0194 (8)	0.0009 (7)	0.0074 (7)	−0.0017 (8)
O3	0.0214 (8)	0.0352 (10)	0.0180 (7)	0.0013 (8)	−0.0035 (6)	0.0083 (8)
O4	0.0424 (12)	0.0860 (19)	0.0466 (12)	−0.0228 (12)	−0.0057 (9)	0.0414 (13)

### Geometric parameters (Å, º)

**Table:**

C1—C2	1.384 (3)	C7—C12A	1.388 (4)
C1—C3^i^	1.384 (3)	C9—C10	1.355 (4)
C1—H1	0.9500	C9—C8B	1.379 (4)
C2—C3	1.387 (3)	C9—C8A	1.379 (4)
C2—C5	1.494 (3)	C9—H9	0.9500
C3—C4	1.485 (3)	C10—F2	1.346 (3)
C4—O1	1.194 (3)	C10—C11	1.367 (4)
C4—N1	1.389 (3)	C11—C12B	1.377 (3)
C5—O2	1.200 (3)	C11—C12A	1.377 (3)
C5—N1	1.384 (3)	C11—H11	0.9500
C6—O4	1.177 (3)	C8A—F1A	1.331 (3)
C6—O3	1.402 (3)	C12A—H12A	0.9500
C6—C7	1.472 (3)	C8B—H8B	0.9500
C7—C8B	1.381 (3)	C12B—F1B	1.315 (10)
C7—C8A	1.381 (3)	N1—O3	1.381 (2)
C7—C12B	1.388 (4)		
			
C2—C1—C3^i^	114.5 (2)	C10—C9—H9	121.3
C2—C1—H1	122.8	C8A—C9—H9	121.3
C3^i^—C1—H1	122.8	F2—C10—C9	118.3 (2)
C1—C2—C3	122.23 (19)	F2—C10—C11	118.5 (3)
C1—C2—C5	129.0 (2)	C9—C10—C11	123.3 (2)
C3—C2—C5	108.78 (19)	C10—C11—C12B	118.2 (2)
C1^i^—C3—C2	123.3 (2)	C10—C11—C12A	118.2 (2)
C1^i^—C3—C4	128.0 (2)	C10—C11—H11	120.9
C2—C3—C4	108.65 (19)	C12A—C11—H11	120.9
O1—C4—N1	126.2 (2)	F1A—C8A—C9	118.1 (2)
O1—C4—C3	130.0 (2)	F1A—C8A—C7	119.4 (2)
N1—C4—C3	103.76 (19)	C9—C8A—C7	122.5 (2)
O2—C5—N1	126.1 (2)	C11—C12A—C7	121.3 (2)
O2—C5—C2	130.6 (2)	C11—C12A—H12A	119.3
N1—C5—C2	103.36 (19)	C7—C12A—H12A	119.3
O4—C6—O3	121.1 (2)	C9—C8B—C7	122.5 (2)
O4—C6—C7	130.0 (2)	C9—C8B—H8B	118.8
O3—C6—C7	108.8 (2)	C7—C8B—H8B	118.8
C8B—C7—C12B	117.4 (2)	F1B—C12B—C11	112.2 (13)
C8A—C7—C12A	117.4 (2)	F1B—C12B—C7	126.4 (13)
C8B—C7—C6	119.9 (2)	C11—C12B—C7	121.3 (2)
C8A—C7—C6	119.9 (2)	O3—N1—C5	122.00 (19)
C12B—C7—C6	122.7 (2)	O3—N1—C4	122.6 (2)
C12A—C7—C6	122.7 (2)	C5—N1—C4	115.35 (19)
C10—C9—C8B	117.4 (2)	N1—O3—C6	111.96 (18)
C10—C9—C8A	117.4 (2)		
			
C3^i^—C1—C2—C3	0.5 (4)	C10—C9—C8A—F1A	−179.5 (3)
C3^i^—C1—C2—C5	179.9 (2)	C10—C9—C8A—C7	−0.5 (5)
C1—C2—C3—C1^i^	−0.5 (4)	C12A—C7—C8A—F1A	179.5 (3)
C5—C2—C3—C1^i^	180.0 (2)	C6—C7—C8A—F1A	0.1 (4)
C1—C2—C3—C4	178.0 (2)	C12A—C7—C8A—C9	0.4 (4)
C5—C2—C3—C4	−1.6 (3)	C6—C7—C8A—C9	−179.0 (3)
C1^i^—C3—C4—O1	1.0 (4)	C10—C11—C12A—C7	0.5 (4)
C2—C3—C4—O1	−177.4 (3)	C8A—C7—C12A—C11	−0.4 (4)
C1^i^—C3—C4—N1	178.1 (2)	C6—C7—C12A—C11	179.0 (3)
C2—C3—C4—N1	−0.2 (2)	C10—C9—C8B—C7	−0.5 (5)
C1—C2—C5—O2	3.1 (4)	C12B—C7—C8B—C9	0.4 (4)
C3—C2—C5—O2	−177.4 (2)	C6—C7—C8B—C9	−179.0 (3)
C1—C2—C5—N1	−176.8 (2)	C10—C11—C12B—F1B	177.2 (15)
C3—C2—C5—N1	2.7 (2)	C10—C11—C12B—C7	0.5 (4)
O4—C6—C7—C8B	−2.9 (5)	C8B—C7—C12B—F1B	−176.7 (17)
O3—C6—C7—C8B	176.6 (3)	C6—C7—C12B—F1B	2.7 (18)
O4—C6—C7—C8A	−2.9 (5)	C8B—C7—C12B—C11	−0.4 (4)
O3—C6—C7—C8A	176.6 (3)	C6—C7—C12B—C11	179.0 (3)
O4—C6—C7—C12B	177.8 (3)	O2—C5—N1—O3	−6.0 (4)
O3—C6—C7—C12B	−2.7 (4)	C2—C5—N1—O3	173.86 (19)
O4—C6—C7—C12A	177.8 (3)	O2—C5—N1—C4	177.1 (2)
O3—C6—C7—C12A	−2.7 (4)	C2—C5—N1—C4	−3.0 (3)
C8B—C9—C10—F2	−179.2 (3)	O1—C4—N1—O3	2.6 (4)
C8A—C9—C10—F2	−179.2 (3)	C3—C4—N1—O3	−174.73 (19)
C8B—C9—C10—C11	0.6 (5)	O1—C4—N1—C5	179.4 (2)
C8A—C9—C10—C11	0.6 (5)	C3—C4—N1—C5	2.1 (3)
F2—C10—C11—C12B	179.2 (3)	C5—N1—O3—C6	99.6 (3)
C9—C10—C11—C12B	−0.5 (5)	C4—N1—O3—C6	−83.7 (3)
F2—C10—C11—C12A	179.2 (3)	O4—C6—O3—N1	−3.5 (4)
C9—C10—C11—C12A	−0.5 (5)	C7—C6—O3—N1	176.9 (2)

Symmetry code: (i) −*x*+1, −*y*, −*z*+1.

### Hydrogen-bond geometry (Å, º)

**Table:**

*D*—H···*A*	*D*—H	H···*A*	*D*···*A*	*D*—H···*A*
C1—H1···O2^ii^	0.95	2.37	3.271 (3)	158
C9—H9···F1*A*^iii^	0.95	2.51	3.287 (3)	139
C9—H9···F2^iv^	0.95	2.63	3.307 (3)	129
C11—H11···O1^v^	0.95	2.39	3.200 (3)	143

Symmetry codes: (ii) −*x*+1, *y*−1/2, −*z*+1/2; (iii) −*x*, −*y*, −*z*; (iv) −*x*, *y*−1/2, −*z*−1/2; (v) *x*, −*y*+3/2, *z*−1/2.
